# RNAome sequencing delineates the complete RNA landscape

**DOI:** 10.1016/j.gdata.2015.07.002

**Published:** 2015-07-11

**Authors:** Kasper W.J. Derks, Joris Pothof

**Affiliations:** Department of Genetics, Erasmus University Medical Center, P.O. Box 2040, 3000 CA, Rotterdam, The Netherlands

**Keywords:** Strand-specific RNA-sequencing, RNAome, Whole transcriptome, RNA expression, RNA abundance

## Abstract

Standard RNA expression profiling methods rely on enrichment steps for specific RNA classes, thereby not detecting all RNA species. For example, small and large RNAs from the same sample cannot be sequenced in a single sequence run. We designed RNAome sequencing, which is a strand-specific method to determine the expression of small and large RNAs from ribosomal RNA-depleted total RNA in a single sequence run. RNAome sequencing quantitatively preserves all RNA classes. This characteristic allows comparisons between RNA classes, thereby facilitating relationships between different RNA classes. Here, we describe in detail the experimental procedure associated with RNAome sequencing published by Derks and colleagues in RNA Biology (2015) [1]. We also provide the R code for the developed Total Rna Analysis Pipeline (TRAP), an algorithm to analyze RNAome sequencing datasets (deposited at the Gene Expression Omnibus data repository, accession number GSE48084).

**Table:** 

Specifications	
Organism/cell line/tissue	*Mus musculus* embryonic stem cells
Sex	N/A
Sequencer or array type	HiSeq2000
Data format	Raw data: sra, and processed: reads per genomic location
Experimental factors	Cisplatin treated (2.7 μM) vs untreated controls
Experimental features	RNA sequencing method of rRNA depleted samples; RNA class comparison; small and large RNAs discovery
Consent	N/A
Sample source location	Department of Genetics, Erasmus MC, Rotterdam, The Netherlands

## 1. Direct link to deposited data

Deposited data: http://www.ncbi.nlm.nih.gov/geo/query/acc.cgi?acc=GSE48084

## 2. Experimental design, materials and methods

### 2.1 Total RNA isolation

**Fig. 1:**
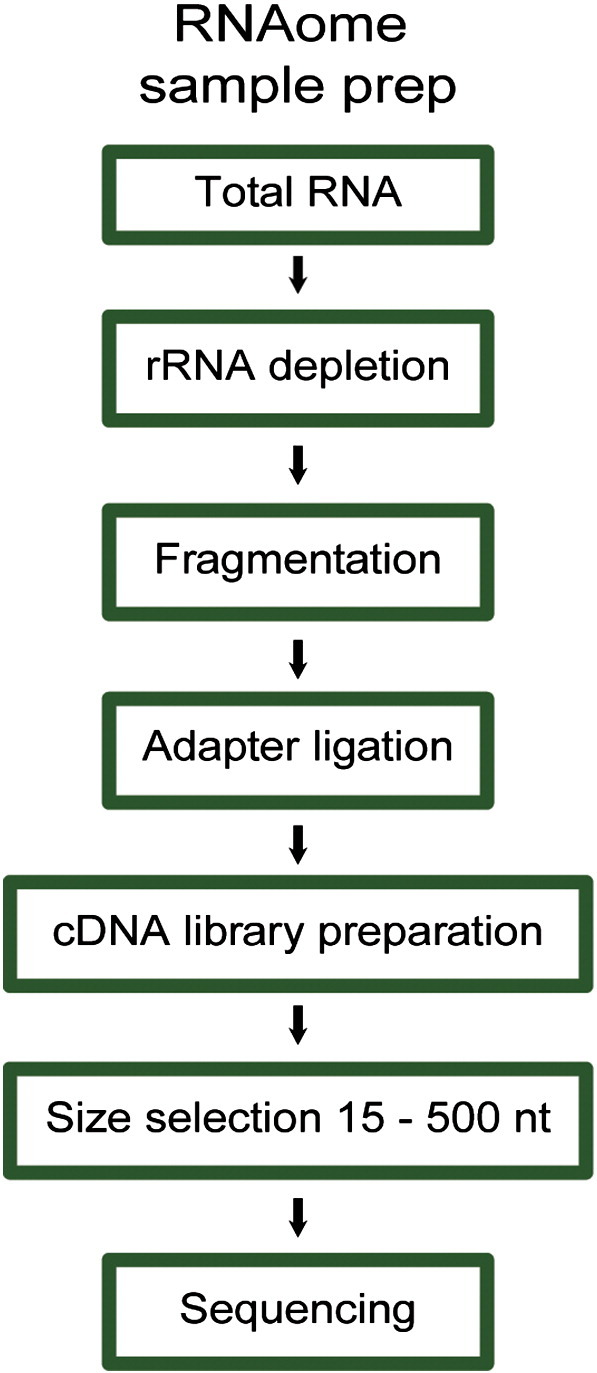
RNAomeSeq sample preparation schematic. 1) rRNA depletion of total RNA. 2) RNA fragmentation, 3) adapter ligation, 4) cDNA library preparation, 5) size selection on gel, 6) Illumina HiSeq2000.

Mouse embryonic stem (mES) cells (HM1) were cultured as described [2]. One vial of mES cells was thawed and grown for 2 passages on feeder-coated plates followed by one passage on gelatin-coated plates. Total RNA was isolated using Qiazol Lysis Reagent (Qiagen) and total RNA was purified using the miRNeasy kit (Qiagen) according to manufacturer's protocols. RNA integrity was determined using the Agilent 2100 Bioanalyzer according to manufacturer's protocol (Fig. 1, row 1 (all scores > 9.0)). This complete procedure was repeated 4 times to obtain 4 independent biological replicates. These samples were used for RNAome sequencing as well as standard poly(A)+-sequencing (Illumina TruSeq mRNA), standard small RNA sequencing (Illumina TruSeq smallRNA v1.5) and Affymetrix gene expression arrays (HT Mouse Genome 430 PM), the latter three methods all serve as controls for RNAome sequencing [1] and were all performed according to manufacturer's protocol.

### 2.2 RNAomeSeq sample preparation

**Fig. 2:**
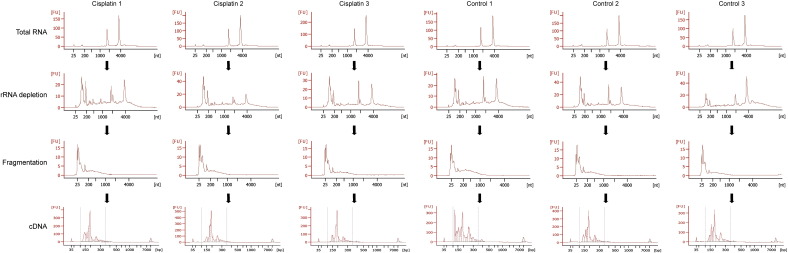
Agilent 2100 Bioanalyzer analysis monitoring 3 steps in the RNAome protocol. Row 1) total RNA isolation, row 2) rRNA depletion, row 3) rRNA fragmentation, row 4) cDNA library.

**Fig. 3:**
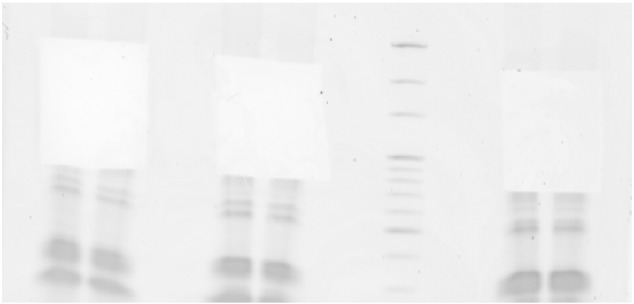
Size selection on gel and adapter-dimer removal. Gels were cut from nucleotide ~ 160 to ~ 645 to remove adapter dimers (~ 145 nucleotides) followed by isolation of adapter-RNA-adapter containing cDNA ranging from 15–500 nt in length.

Ribosomal RNA (rRNA) depletion was performed using the RiboMinus Eukaryote Kit (Life Science) according to manufacturer's protocol. 10 μg of total RNA was incubated with biotin-labeled LNA probes (2 for each of the 4 rRNA species, i.e. 5 S, 5.8 S, 18 S and 28 S) and hybridized to streptavidin-coated magnetic beads. rRNA-depleted samples were concentrated using the RiboMinus Concentration Module according to manufacturer's protocols. Concentrated rRNA-depleted samples were fragmented by sonication (Covaris s200, duty cycle 5% and 200 burst/cycle for 210 s), resulting in fragments smaller than 500 nucleotides. Agilent 2100 Bioanalyzer analysis shows that rRNA depletion and fragmentation of the rRNA-depleted RNA were highly reproducible (Fig. 1, rows 2–3). The cDNA library was generated using the small RNASeq kit (Illumina TruSeq smallRNA v1.5) according to the manufacturer's protocol. In short, specific bar-coded adapters were ligated to the fragmented rRNA-depleted samples followed by reverse transcriptase and amplification for 11 cycles. The cDNA library was fractionated on a 15% Tris-borate-EDTA. Adapter dimers, approx. 145 nucleotides in length, were removed by excising RNAs ranging 160–645 nucleotides of length (Fig. 2). The excised gel containing the adapter-ligated cDNA fragments, corresponding to RNAs 15–500 nt in length, were extracted from the gel using the gel breaker kit (IST Engineering) (Fig. 3). Finally, cDNA libraries were pooled after extraction and further prepared for sequencing.

### 2.3 Sequencing

Pooled cDNA libraries consisted of equal concentration bar-coded samples, i.e., 3 mock- and 3 cisplatin-treated samples and were sequenced in 2 lanes, 36 bp single read on the Illumina HiSeq2000. This yielded on average 52 million sequence reads per sample. The dataset has been deposited at the Gene Expression Omnibus database, GSE48084.

### 2.4 Total RNA analysis pipeline

**Fig. 4:**
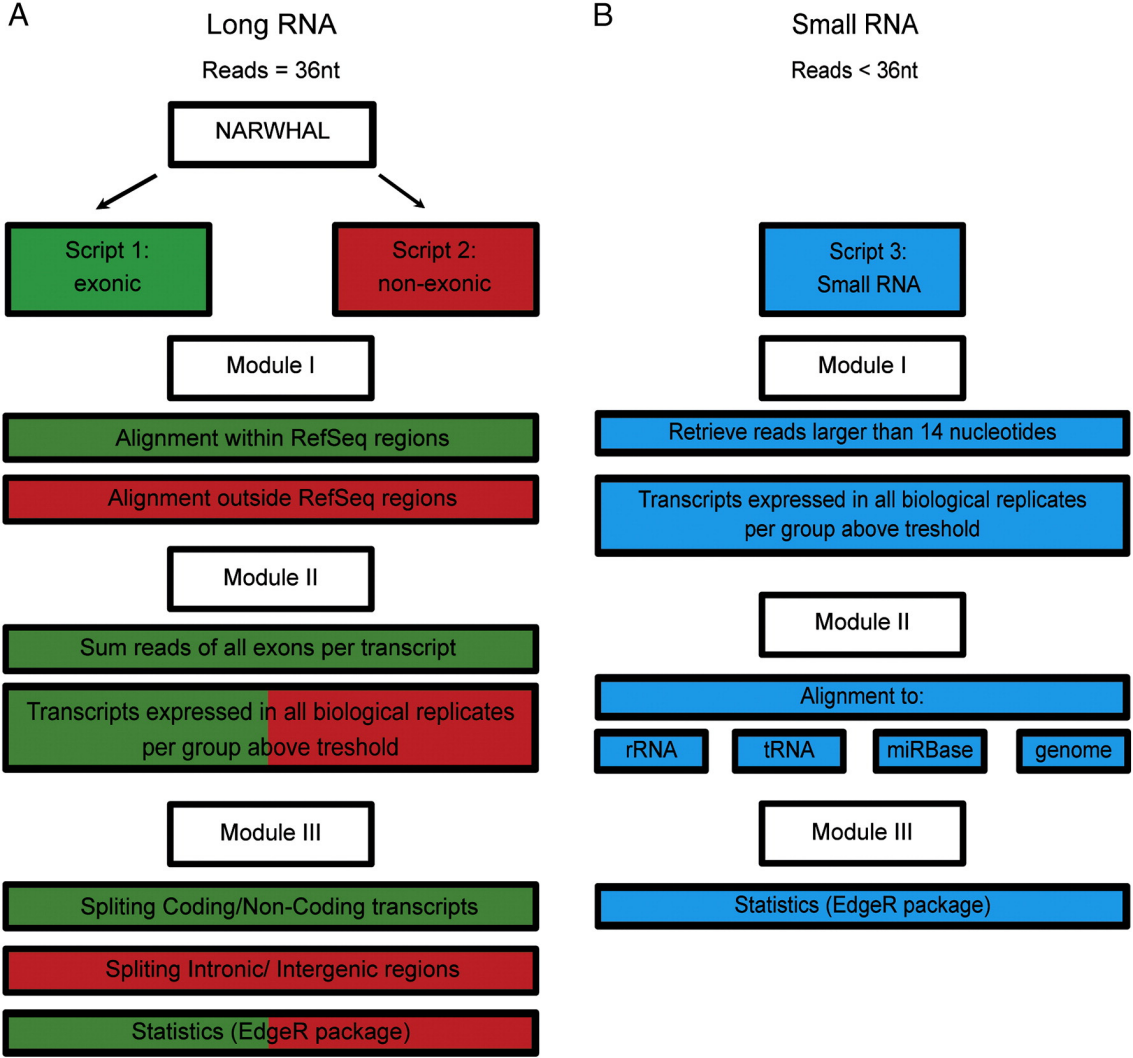
Schematic of the Total RNA Analysis Pipeline, TRAP for analysis of sequencing datasets. (A) Modules for long RNA analysis, script 1 (green) for RefSeq annotated exonic transcripts and script 2 (red) for RefSeq annotated non-exonic regions. (B) Modules for small RNA analysis, script 3 (blue) to sequentially align trimmed reads to rRNA, tRNA fragments and the microRNA database (miRBase version 21).

The analysis of the sequencing dataset was performed with TRAP, which refers to Total RNA Analysis Pipeline (Fig. 4). The analysis was performed on a quad-core CPU desktop with 64-bits windows system and 16 gigabyte RAM. The reads were, prior to the analysis with TRAP, trimmed for adapter sequences. 36 nt reads in length were aligned to the reference mouse genome (mm9 build) using Tophat (version 1.3.1.Linux_x86_64, − coverage-search, − butterfly-search, − segment-mismatches 1, –segment-length 18) via the NARWHAL automation software (Fig. 4A) [3]. TRAP uses several R Bioconductor [4] packages, e.g., Biostrings (version 2.26.3), Rsamtools (version 1.10.2), IRanges (version 1.16.6), GenomicRanges (version 1.10.7), Limma [5] and EdgeR [6]. Reads that aligned within and between RefSeq transcripts were extracted from the resulting BAM files using Scripts 1 and 2 in module I (Fig. 4A). RefSeq can be replaced in TRAP by other annotations such as GENCODE depending on the user's preference. Exonic reads were summed per transcript. In module II, a specific transcript or region was referred to as expressed, when a minimum number of 1 read aligned to a transcript or non-exonic region across all biological replicates in at least one of the experimental groups (Fig. 4A). In module III, expressed transcripts were divided by RefSeq identifiers into coding (NM identifier) and non-coding transcripts (NR identifier) (Fig. 4A). The non-exonic regions were divided by location into an intergenic or intronic category. TRAP's default setting uses the statistical algorithm EdgeR [6], because it was the best performing statistical algorithm on our dataset [1]. Other statistical algorithms can easily replace EdgeR and implemented into TRAP, depending on the experimental set-up/dataset used.

Next, TRAP can be used to analyze reads smaller than 36 nucleotides (Fig. 4B). In module I, trimmed sequence reads smaller than 14 nucleotides of length were discarded. Reads were referred to as expressed when reaching a specific threshold, which was defined as a minimal of 1 read being present in all biological replicates in at least one experimental group. In Module II, the expressed reads were first aligned to rRNA sequences (5 s and 5.8 s), tRNA sequences, the miRBase [7] database (v21) (using vmatchPattern from the Biostrings package) or the genome (using NARWHAL [3], using only bowtie; − best, − l 32, − n 2, − M 1) (Fig. 4B). In module III, statistical analysis of the tRNA aligned reads and miRBase [7] aligned reads (microRNAs) was performed with EdgeR [6]. (Fig. 4B) The reads aligned to the genome (small RNAs) were further processed as long RNAs in Scripts 1 and 2 in TRAP. The thresholds in TRAP can be manually set and adjusted according to the users' preference. The R code for TRAP can be found on our website (http://rna-ome.erasmusmc.nl/). Note that TRAP can also be used for standard poly(A)+-sequencing, standard small RNA sequencing.

## Funding

This work was supported by The Netherlands Toxicogenomics Center (NTC), and National Institute of Health (NIH)/National Institute of Aging (NIA) (1PO1 AG-17242-02), NIEHS (1UO1 ES011044).
